# Volatilome and Bioaccessible Phenolics Profiles in Lab-Scale Fermented Bee Pollen

**DOI:** 10.3390/foods10020286

**Published:** 2021-01-31

**Authors:** Pasquale Filannino, Raffaella Di Cagno, Giuseppe Gambacorta, Ali Zein Alabiden Tlais, Vincenzo Cantatore, Marco Gobbetti

**Affiliations:** 1 Department of Soil, Plant and Food Science, University of Bari Aldo Moro, 70126 Bari, Italy; giuseppe.gambacorta@uniba.it (G.G.); vincenzo.cantatore@uniba.it (V.C.); 2 Faculty of Sciences and Technology, Libera Università di Bolzano, 39100 Bolzano, Italy; AliZeinAlabiden.Tlais@natec.unibz.it (A.Z.A.T.); marco.gobbetti@unibz.it (M.G.)

**Keywords:** pollen, fermentation, lactic acid bacteria, volatile compounds, phenolics, flavonoids

## Abstract

Bee-collected pollen (BCP) is currently receiving increasing attention as a dietary supplement for humans. In order to increase the accessibility of nutrients for intestinal absorption, several biotechnological solutions have been proposed for BCP processing, with fermentation as one of the most attractive. The present study used an integrated metabolomic approach to investigate how the use of starter cultures may affect the volatilome and the profile of bioaccessible phenolics of fermented BCP. BCP fermented with selected microbial starters (Started-BCP) was compared to spontaneously fermented BCP (Unstarted-BCP) and to unprocessed raw BCP (Raw-BCP). Fermentation significantly increased the amount of volatile compounds (VOC) in both Unstarted- and Started-BCP, as well as modifying the relative proportions among the chemical groups. Volatile free fatty acids were the predominant VOC in Unstarted-BCP. Started-BCP was differentiated by the highest levels of esters and alcohols, although volatile free fatty acids were always prevailing. The profile of the VOC was dependent on the type of fermentation, which was attributable to the selected *Apilactobacillus kunkeei* and *Hanseniaspora uvarum* strains used as starters, or to the variety of yeasts and bacteria naturally associated to the BCP. Started-BCP and, to a lesser extent, Unstarted-BCP resulted in increased bioaccessible phenolics, which included microbial derivatives of phenolic acids metabolism.

## 1. Introduction

Bee-collected pollen (BCP) is currently receiving increasing attention due to its remarkable levels of valuable nutrients and bioactive compounds [1,2,3,4]. BCP is rich in proteins (5–60%), essential amino acids, simple sugars (13–55%), unsaturated and saturated fatty acids (1–10%), and crude fibre (0.3–20%). Other minor constituents include minerals (Ca, Mg, Fe, Zn, Cu, and with a high K/Na ratio), vitamins (provitamin A, vitamin E, niacin, thiamine, biotin and folic acid), phenolics (flavonoids and phenolic acids), carotenoid pigments, and phytosterols [2]. According to Denisow and Denisow-Pietrzyk [2], fifteen grams of BCP may account for a significant part of the required daily intake for sugars, proteins, and some vitamins and minerals. Even though BCP is mainly exploited as a dietary supplement, it is not easily digestible by humans, and more than 50% of its nutrients are not bioaccessible due to the intine–exine complex of pollen grain wall. The exine mostly consists of sporopollenin, which provides strong chemical and physical resistances and protects the compounds inside the pollen grains [3,4,5]. Therefore, BCP needs to be processed before human consumption, in order to impair the pollen wall and to increase the accessibility of nutrients for intestinal absorption. Several biotechnological options have been proposed for BCP processing, including physical, chemical and biological treatments [6,7,8,9,10]. Fermentation is one of the most attractive opportunities, because it emulates the natural maturation process of bee bread, which is mediated by bee-associated microbial communities [11]. Within the beehive, the conversion of BCP to bee bread results from several biochemical changes, including the modification of the intine-exine complex, which in turn leads to an increased accessibility of nutrients and bioactive compounds [3,4]. The predominance of lactic acid bacteria in bee bread was previously reported, with *Apilactobacillus kunkeei*, *Fructobacillus fructosus*, and *Lactiplantibacillus plantarum* as dominant species. The occurrence of yeasts, molds, and other aerobic and anaerobic bacteria has also been described. Many of these microorganisms are metabolically relevant during bee bread maturation, while some of them are simply contaminants [12,13,14,15]. Due to the prominent role of this complex microbial consortium, the emulation of bee bread fermentation through a controlled lab-scale process is difficult. Several studies investigated the aptitude of selected microbial starters to ferment BCP in order to obtain dietary supplements with improved nutritional and functional features compared to the raw BCP [16,17,18]. During BCP fermentation, innumerable biochemical reactions are mediated by the primary and secondary metabolisms of microorganisms. Such metabolic pathways frequently are connected to the adaptive growth and survival of microorganisms [19]. Most importantly, some microbial metabolic traits may modify the bioaccessibility and bioavailability of phytochemicals. Other than taking part in breakdown of pollen walls, microorganisms may drive the degradation of complex molecules, which in turn release smaller molecules often characterized by greater bioaccessibility and bioavailability. For instance, free volatile terpenes may be released upon hydrolysis of glycosidically conjugated precursors. Similarly, microbial enzymes may contribute to the release of proteins- and carbohydrates-associated phenolics. To the best of our knowledge, there is a lack of data on the metabolite changes during BCP fermentation, especially with regard phenolics and other secondary vegetables metabolites. This study proposed an integrated metabolomic approach to investigate how the use of starters may affect the volatilome and the profile of bioaccessible phenolics during fermentation of BCP. In addition to provide information about changes in some phytochemicals bioaccessibility, profiling of volatilome may enhance the understanding of microbial community dynamics and contribute to the ad-hoc selection of starters for BCP processing. Furthermore, volatilome analyses identifies flavor constituents affecting the consumer’s acceptability. Under the condition of our study, the effects of spontaneous fermentation of BCP were compared to that of a selected consortium composed by *Apilactobacillus kunkeei* strains and *Hanseniaspora uvarum* [13]. *A. kunkeei* was chosen, since strongly associated to the alimentary tract and to the stored food of honeybees [13,20]. An ecological role was previously proposed for *H. uvarum* during BCP fermentation in consortium with *A. kunkeei* [13].

## 2. Materials and Methods

### 2.1. Microorganisms and Culture Conditions

*A. kunkeei* PF12, PL13, and PF15 (formerly *Lactobacillus kunkeei*) and *H. uvarum* AN8Y27B belonging to the Culture Collection of the Department of Soil, Plant and Food Science, University of Bari Aldo Moro (Bari, Italy), were used as mixed starters for BCP fermentation, according to the protocol designed by Di Cagno et al. [13]. *A. kunkeei* strains were routinely cultured at 30 °C for 24 h in fructose-yeast extract-polypeptone (FYP) broth (D-fructose 10 g L^−1^, yeast extract 10 g L^−1^, polypeptone 5 g L^−1^, sodium acetate 2 g L^−1^, Tween 80 0.5 g L^−1^, MgSO_4_·7H_2_O 0.2 g L^−1^, MnSO_4_·4H_2_O 0.01 g L^−1^, FeSO_4_·7H_2_O 0.01 g L^−1^, NaCl 0.01 g L^−1^). *H. uvarum* AN8Y27B was cultivated at 30 °C for 36 h in yeast extract-peptone-dextrose (YPD) broth (yeast extract 10 g L^−1^, bacteriological peptone 20 g L^−1^, and dextrose 20 g L^−1^).

### 2.2. Fermentation of BCP

Bee-collected pollen (BCP) originated from flowers of ivy (*Hedera helix* L.) and was collected through pollen traps from different hives located in organic fields. After collection, BCP was mixed, stored at 4 °C in sterile glass containers, and processed within 24 h of collection. BCP was fermented according to the protocol designed by Di Cagno et al. [13]. Briefly, *A. kunkeei* strains and *H. uvarum* AN8Y27B were cultivated until the late exponential growth phase was reached. In order to identify the late exponential growth phase, growth kinetics were previously modelled by monitoring the microbial growth through plate count of *H. uvarum* on Sabouraud Dextrose Agar (Oxoid, Dublin, Ireland) and of *A. kunkeei* strains on Fructose Yeast extract Polypeptone agar [13]. Microbial cells were washed twice in 50 mM phosphate buffer (pH 7.0) and inoculated into BCP at the final density of ca. 8 Log CFU g^−1^. BCP was added with sterile water to reach the final water content of 40% (*w w*^−1^), placed into sealed tubes, and incubated at 30 °C for 216 h. BCP fermented by the selected mixed starter (Started-BCP) was characterized as described below. BCP treated under the same conditions except for the use of microbial starters (Unstarted-BCP), and fresh BCP without any treatment (Raw-BCP) were used as controls. Fermentation was monitored by measuring the total titratable acidity (TTA). Briefly, 10 g of BCP was homogenized with 90 mL of distilled water through a Classic Blender 400 (PBI International, Milan, Italy), and the TTA was expressed as the amount (mL) of NaOH 0.1 M necessary to achieve a pH of 8.3.

### 2.3. Volatilome Analysis

Volatile compounds (VOC) were investigated through HS-SPME-GC-MS as described by Gambacorta et al. [21], with few modifications. A Trace 1300 gas chromatograph (Thermo Fisher Scientific, Rodano, Italy) was used with a VF-WAXms capillary column 60 m length × 0.25 mm I.D. × 0.25 μm film (Agilent, Santa Clara, CA, USA), and an ISQ single quadrupole mass spectrometer (Thermo Fisher Scientific). The extraction was performed through a TriPlus RSH™ Autosampler (Thermo Fisher Scientific), by using a DVB-CAR-PDMS fiber (Supelco, Bellefonte, PA, USA). After conditioning for 10 min at 40 °C the sample (1 g) and 10 µL of 2-octanol (internal standard, 81.9 ng L^−1^ in water) in 20 mL screw-cap vial with a PTFE-silicon septum, the extraction was undertaken for 40 min at 40 °C. After that, desorption of volatiles from fiber took place in a spitless mode for 3 min at 220 °C.

The chromatographic conditions were: oven, 45 °C (5 min) to 210 °C at 4 °C/min, held for 3 min; detector, source temperature 250 °C; transfer line temperature 250 °C; carrier gas, helium at constant flow of 0.4 mL min^−1^. The impact energy was 70 eV. Data were acquired using the full-scan mode in the range of 35 to 150 m/z at an acquisition rate of 7.2 Hz. Tentatively identification of the VOC was carried out by comparing the experimental spectra with those reported in the NIST Library and with those obtained by the available pure standard compounds. Volatiles were quantified using relative areas related to the 2-octanol as the internal standard and expressed as ppb. Data acquisition was interfaced to a computer workstation running Xcalibur v 4.1 software (Thermo Fisher Scientific).

### 2.4. In Vitro Gastrointestinal Batch Digestion

To evaluate the bioaccessibility of bioactive compounds in the BCP samples, we chose phenolics as target compounds. Phenolics bioaccessibility in Raw-, Unstarted-, and Started-BCP was investigated through an in vitro gastrointestinal batch digestion process that was carried out as described by Eid et al. [22] and Celep et al. [23], with few modifications. Ten g of freeze-dried BCP samples were added to 50 mL of distilled water and mixed in a stomacher for 2 min. Then, the solution was mixed with α-amylase from human saliva (Sigma-Aldrich, Steinheim, Germany) (20 mg) in CaCl_2_ (1 mM, 6.25 mL) and incubated at 37 °C for 30 min under a stirring condition (100 rpm) to mimic the oral digestion phase. To simulate gastric digestion, pepsin from pig gastric mucosa (Sigma-Aldrich) (2.7 g) was dissolved in 25 mL 0.1 M HCl and added to mixture. Then, the pH value was adjusted to 2.0 using 6 M HCl and the sample was incubated at 37 °C for 3 h under a stirring condition. To simulate small intestine conditions, pancreatin from porcine pancreas (Sigma-Aldrich) (560 mg) and bile from porcine (Sigma-Aldrich) (3.5 g) were dissolved in 125 mL of 0.1 M NaHCO_3_ and added to the sample. The pH value was slowly adjusted to 7.0 by using 6 M NaOH and a segment of cellulose dialysis tubing (molecular weight cut off 12 kDa) was placed inside the beaker. The semipermeable cellulose membrane was used as a simplified model of the epithelial barrier [24,25]. The mixture was incubated at 37 °C for 3 h under a stirring condition (100 rpm). After the incubation, the solution that diffused into the dialysis tubing was taken as the bioaccessible fraction. The latter was centrifuged at 12,888× *g*, and filtered through a nylon syringe filter with a pore size of 0.45 μm.

### 2.5. Analyses of Bioaccessible Phenolics

Acetonitrile (CH_3_CN, LC-MS grade) and methanol (CH_3_OH, LC-MS grade) were from Sigma-Aldrich. LC-MS grade formic acid (HCOOH) was from Fluka Sigma-Aldrich (Milan, Italy) and Milli-Q water from Millipore system (Millipore, Billerica, MA, USA). Commercial standards (Sigma-Aldrich) allowed the identification and quantification of phenolic compounds.

A phenolics profile of the bioaccessible fraction was investigated through the LC-ESI-MS/MS as described by Tlais et al. [26]. A UHPLC Dionex 3000 (Thermo Fisher Scientific., Germering, Germany), was equipped with a TSQ Quantum™ Access MAX Triple Quadrupole Mass Spectrometer (Thermo Fisher Scientific., Germering, Germany) and an electrospray source. Phenolic compounds were separated through a Waters Acquity HSS T3 column (1.8 μm, 100 × 2.1 mm) (Milford, MA, USA). Eluent A consisted of 0.1% (*v*·*v*^−1^) formic acid in water, and eluent B consisted of 0.1% (*v*·*v*^−1^) formic acid in acetonitrile. Sample (3 μL) was eluted with the following gradient: 0.0–3.0 min from 2% to 20% B, 3.0–4.3 min at 20% B, 4.3–9 min from 20% to 45% B, 9–11 min from 45% to 100% B, 11–13 min at 100%, and 13–15 min from 100% to 5% B. Elution was at 40 °C, with a flow rate maintained at 0.4 mL/min.

The mass spectrometer was operated in positive and negative ionization modes using the following conditions: sheath gas at 30 (arbitrary units), ion sweep gas pressure at 0, aux valve flow at 10 (arbitrary units), aux temperature 250 °C, spray voltage at 3.5 kV in positive mode and −3 kV in negative mode, and capillary temperature at 350 °C. Collision energy values optimized for each compound were reported in Table S1. Target compounds were identified based on their reference standard, retention time, qualifier and quantifier ion (Tables S1 and S2). Calibration curves were obtained with selected chemical standards and results were expressed as mg per 100 g BCP, after normalization with phloridzin as the internal standard.

Separation, identification and quantification of microbial derivatives of phenolic acids were performed as described by Filannino et al. [27]. The Ultimate 3000 HPLC system (Dionex, Germering, Germany) was equipped with a photodiode array detector (PAD 3000), a low-pressure pump Ultimate 3000, and an injector loop Rheodyne (Rheodyne, USA, volume 20 μL). Phenolic compounds were separated through a Kinetex C18 Phenomenex (150 × 4.6 mm with a particle size of 5 μm) column (Thermo Fisher Scientific). Eluent A consisted of 0.1% (*v·v*^−1^) trifluoroacetic acid in water, and eluent B consisted of 0.1% (*v·v*^−1^) trifluoroacetic acid in acetonitrile. Sample (10 μL) was eluted with the following gradient: 0.0–5.0 min from 5% to 10% (B), 5.0–25 min from 10% to 40% (B), 25–45 min from 40% to 90% (B), 90% (B) for 5 min, and 45–50 min from 90% to 5% (B). Elution was at 35 °C, with a flow rate maintained at 1.0 mL/min. Absorption spectra were recorded by using a scan mode ranging from 220 to 500 nm absorbances, and phenolic acid derivatives were detected at 280, 310 and 320 nm wavelengths. Instrument calibration was using the concentration range for all standards from 0.1 to 100 mg L^−1^ with R^2^ of more than 0.95. Data were acquired and analyzed by using the Chromeleon Software version 7 (Dionex, Germering, Germany). Results were expressed as mg per 100 g BCP.

### 2.6. Statistical Analysis

Analyses were carried out in triplicate on three biological replicates for each condition. Data were subjected to an analysis of variance (ANOVA) test for multiple comparisons (one-way ANOVA followed by Tukey’s procedure at *p* < 0.05), using the statistical software, Statistica 7.0 (Statsoft, Hamburg, Germany).

## 3. Results and Discussion

### 3.1. Volatilome Analysis

**Figure 1:**
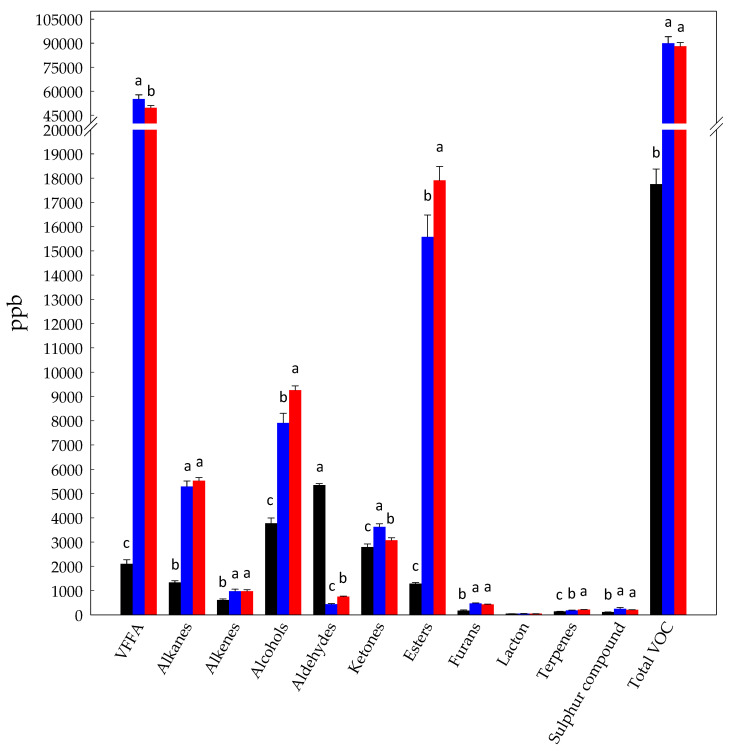
Concentration (ppb) of volatile free fatty acids (VFFA), alkanes, alkenes, alcohols, aldehydes, ketones, esters, furans, lacton (2(3H)-furanone, dihydro-5-methyl-), terpenes, sulphur compound (dimethyl sulfide), and total volatile compounds (total VOC identified in Raw- (black bars), Unstarted- (blue bars), and Started-bee-collected pollen (BCP) (red bars). Data are the means (± SD) of three independent experiments analyzed in triplicate. Data were subjected to one-way analysis of variance (ANOVA); pair-comparison of treatment means was achieved by Tukey’s procedure at *p* < 0.05. For each chemical group, bars with different superscript letters differ significantly (*p* < 0.05).

BCP samples were characterized for the profile of VOC by HS-SPME-GC-MS. VOC (106) were identified and grouped according to the following chemical groups (Figure 1, Table S3): volatile free fatty acids (11 compounds identified), alkanes (9), alkenes (8), alcohols (23), aldehydes (6), ketones (12), esters (27), furans (3), lactons (1), terpenes (4), and sulphur compounds (1). At a glance, the levels of VOC differentiated the BCP samples. Total VOC in Raw-BCP were estimated at 17,751 ± 628 ppb, with aldehydes as predominant group (30% of the total VOC), followed by alcohols (21%), ketones (16%), and volatile free fatty acids (VFFA) (12%). The other chemical groups accounted for the remaining 21% of the total VOC. Few data on BCP volatiles are available in literature. Furthermore, it is not simple to compare our results with any previously obtained, because quantitative and qualitative compositions of VOC are mainly linked to the floral species, and, to a lesser extent, to climatic conditions and geographical locations. Thus, each pollen has its own specific VOC profile. VOC profiles approaching ours were previously found in bee pollen and honey samples [28,29,30,31]. Volatiles are strongly related to some BCP properties. They confer a characteristic basic odor and aroma to BCP, which may impact on consumers acceptability. However, some VOC may exert antimicrobial or antioxidant activities [32,33,34] or act as signaling molecules for insects [35,36].

Under the conditions of our study, Started-BCP underwent controlled fermentation driven by the selected starters composed by three *A. kunkeei* strains and *H. uvarum* AN8Y27B [13]. On the contrary, Unstarted-BCP was subjected to spontaneous fermentation carried out by the autochthonous microbiota naturally associated to BCP. Fermentation significantly (*p* < 0.05) increased the amount of total VOC in both Unstarted- (90,014 ± 4012 ppb) and Started-BCP (88,105 ± 2285 ppb), as well as modifying the relative proportions among the chemical groups (Figure 1). VFFA were the predominant VOC in Unstarted-BCP (61% of total VOC) followed by esters (17%) and alcohols (9%). Similarly, VFFA (56% of total VOC), esters (20%) and alcohols (11%) prevailed in Started-BCP. Comparing BCP samples, the highest (*p* < 0.05) level of aldehydes (5352 ± 60 ppb) distinguished Raw-BCP. Unstarted-BCP showed by the highest (*p* < 0.05) amount of VFFA (55,229 ± 2508 ppb). Esters and alcohols were detected at the highest (*p* < 0.05) concentrations in Started-BCP (17,906 ± 577 and 9260 ± 175 ppb, respectively) (Figure 1).

Main aldehydes identified in Raw-BCP were hexanal (3650 ± 91 ppb), 2,4-heptadienal (E,E) (813 ± 31 ppb), nonanal (633 ± 37 ppb), and propanal (230 ± 11 ppb) (Table S3). Such compounds were totally or mostly depleted during fermentation of BCP (Table S3). It was likely due to the instability of aldehydes, which in fermented matrices frequently undergo reduction to alcohols, or oxidation to carboxylic acids [37]. This could explain the lowest levels (*p* < 0.05) of aldehydes and the highest (*p* < 0.05) amounts of VFFA and alcohols found in Unstarted- and Started-BCP (Figure 1).

**Figure 2:**
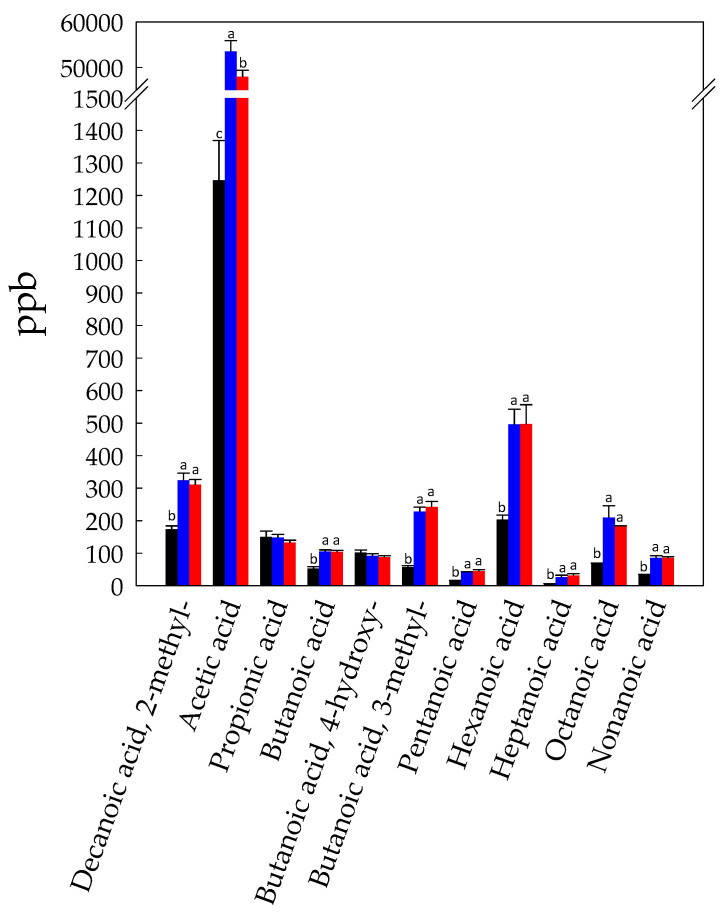
Concentration (ppb) of volatile free fatty acids identified in Raw- (black bars), Unstarted- (blue bars), and Started-BCP (red bars). Data are the means (±SD) of three independent experiments analyzed in triplicate. Data were subjected to one-way ANOVA; pair-comparison of treatment means was achieved by Tukey’s procedure at *p* < 0.05. For each compound, bars with different superscript letters differ significantly (*p* < 0.05).

The levels of VFFA were more than twenty fold higher in Unstarted- and Started-BCP compared to Raw-BCP (Figure 1). Fatty acids are present in pollen both as triacylglycerols and in free form [38]. Acetic acid (1246 ± 122 ppb), hexanoic acid (204 ± 13 ppb), 2-methyldecanoic acid (173 ± 11 ppb), and propionic acid (150 ± 18 ppb) were the VFFA most represented in Raw-BCP (Figure 2). Accumulation of VFFA during BCP fermentation was one of the main microbiota dependent trend observed in our study (Figure 2). The increase was mainly due to the release of acetic acid (Figure 2), which represents, along with lactic acid, one of the main end metabolites of *A. kunkeei* [20]. This is in agreement with the inoculum of *A. kunkeei* selected strains in Started-BCP, which drove the BCP fermentation processes [13]. The high level of acetic acid detected into Unstarted-BCP was likely due to the variety of bacteria naturally associated to the BCP (e.g., Alpha 2.2 bacteria, *A. kunkeei*, Actinobacteria) that may have had a role during spontaneous fermentation of Unstarted-BCP [14,39]. Furthermore. VFFA, like hexanoic acid, 2-methyl-decanoic acid, and 3-methyl-butanoic acid, significantly increased (*p* < 0.05) in both Unstarted- and Started-BCP (Figure 2). This is consistent with the decrease of aldehydes, like hexanal and nonanal, which were likely oxidized to the corresponding acids. We may not exclude a role of the *H. uvarum* AN8Y27B or of the authochthonous yeasts in the VFFA increase. Short and medium fatty acids are produced by yeasts as intermediates in the biosynthesis of long-chain fatty acids for their cellular membrane [40]. The release of VFFA by yeasts is strongly species- and strain-dependent. VFFA may significantly contribute to the BCP flavour, and are usually associated with sweaty, grass, cheesy, fatty, acid, or rancid notes [41]. The increase of VFFA during BCP fermentation was in agreement with the higher (*p* < 0.05) TTA values observed in Started- (135 ± 3 mL NaOH 0.1 M per 10 g of BCP) and Unstarted-BCP (130 ± 4 mL NaOH 0.1 M per 10 g of BCP) compared to Raw-BCP (52 ± 4 mL NaOH 0.1 M per 10 g of BCP).

**Figure 3:**
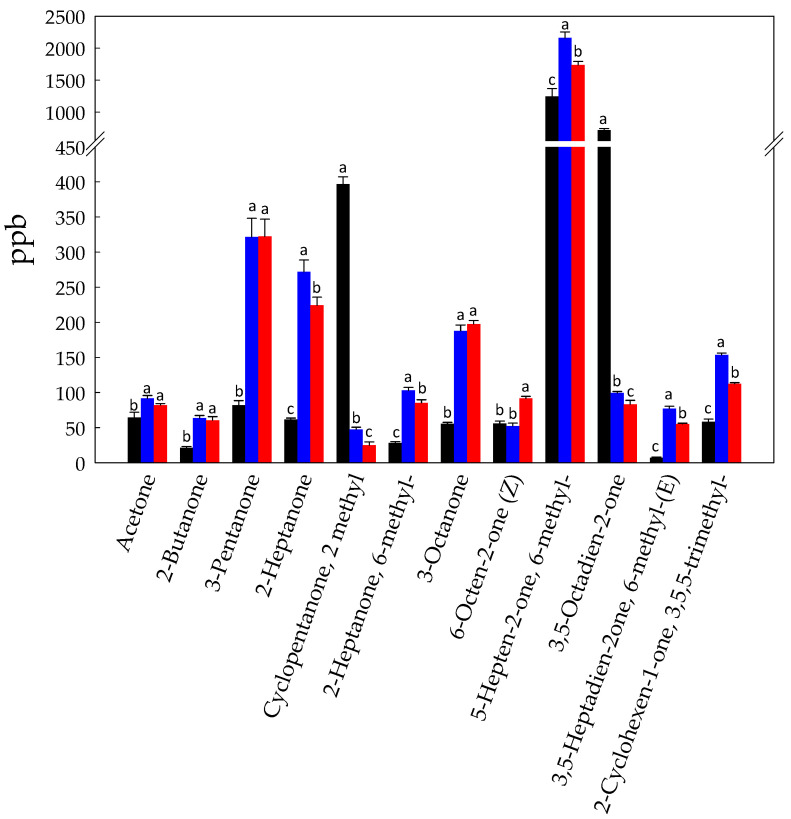
Concentration (ppb) of ketones identified in Raw- (black bars), Unstarted- (blue bars), and Started-BCP (red bars). Data are the means (± SD) of three independent experiments analyzed in triplicate. Data were subjected to one-way ANOVA; pair-comparison of treatment means was achieved by Tukey’s procedure at *p* < 0.05. For each compound, bars with different superscript letters differ significantly (*p* < 0.05).

Ketones may give a noticeable contribution to aroma in food. Six-methyl-5-Hepten-2-one (1244 ± 121 ppb), 3,5-Octadien-2-one (719 ± 25 ppb), and 2 methyl-cyclopentanone (397 ± 10 ppb) were the most abundant in Raw-BCP (Figure 3). The formation of ketones was likely due to the yeasts, especially methyl ketones which arise from incomplete β-oxidation of fatty acids [42,43]. Ketones may either be oxygenated to esters or be reduced to the corresponding alcohols [44,45,46]. Overall, the level of ketones increased in both fermented samples (Figure 3). The highest increase was detected for the 6-methyl-5-hepten-2-one (2158 ± 92 and 1735 ± 57 ppb in Unstarted- and Started-BCP, respectively) (Figure 3). On the other hand, a noticeable decrease of 3,5-Octadien-2-one was also found in fermented BCP samples, with final concentrations of 99 ± 2 and 83 ± 6 ppb in Unstarted- and Started-BCP, respectively.

**Figure 4:**
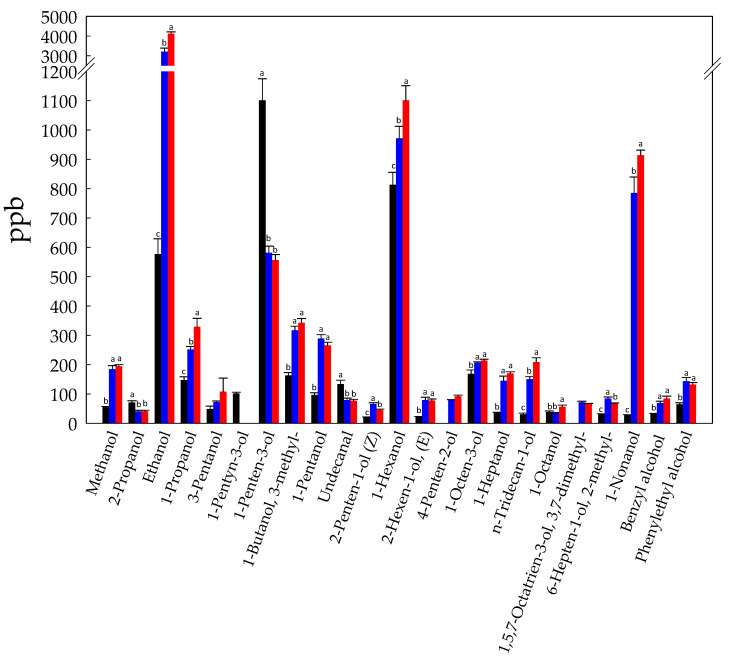
Concentration (ppb) of alcohols identified in Raw- (black bars), Unstarted- (blue bars), and Started-BCP (red bars). Data are the means (± SD) of three independent experiments analyzed in triplicate. Data were subjected to one-way ANOVA; pair-comparison of treatment means was achieved by Tukey’s procedure at *p* < 0.05. For each compound, bars with different superscript letters differ significantly (*p* < 0.05).

1-Penten-3-ol (1101 ± 74 ppb), 1-hexanol (813 ± 42 ppb), and ethanol (576 ± 53 ppb) were the main alcohols detected in Raw-BCP (Figure 4). During fermentation ethanol increased (*p* < 0.05) more than five and seven folds in Unstarted- and Started-BCP, respectively (Figure 4). *A. kunkeei* strains are unable to synthetize ethanol through the 6-phosphogluconate/phosphoketolase pathway, due to the lack of alcohol dehydrogenase activity [20]. Thus, the noticeable production of ethanol may be traced back to the metabolism of *H. uvarum* AN8Y27B or by autochthonous yeasts. 1-Nonanol and 1-hexanol also sharply increased, especially in Started-BCP, and overall, an increasing trend was found during fermentation for most of the detected alcohols (Figure 4). As reported above, alcohols may originate from the reduction of aldehyde. Furthermore, accumulation of branched alcohols (e.g., 3-methyl-1-butanol) may be the consequence of the liberation of free amino acids during BCP fermentation [13]. Free amino acids play a central role as a flavour-forming substrate in lactic acid bacteria and yeasts. In particular, branched alcohols result from branched amino-acid catabolism, which imply conversion of leucine, isoleucine, and valine to the respective α-keto-acids by means of aminotransferases, and subsequent decarboxylation to aldehydes and conversion to alcohols [47,48]. Phenylalanine was also shown to be catabolized by yeasts via the Ehrlich pathway to generate α-keto acid and aldehyde intermediates that are converted to alcohols [49]. Alcohols are important flavor-active metabolites, with the exception of ethanol, which has a neutral odor and does not contribute directly to the overall flavor [50]. 1-Hexanol has been reported to contribute to the sensation of herbaceous and grass. 1-Nonanol and 1-Propanol were correlated to fruity notes [51].

**Figure 5:**
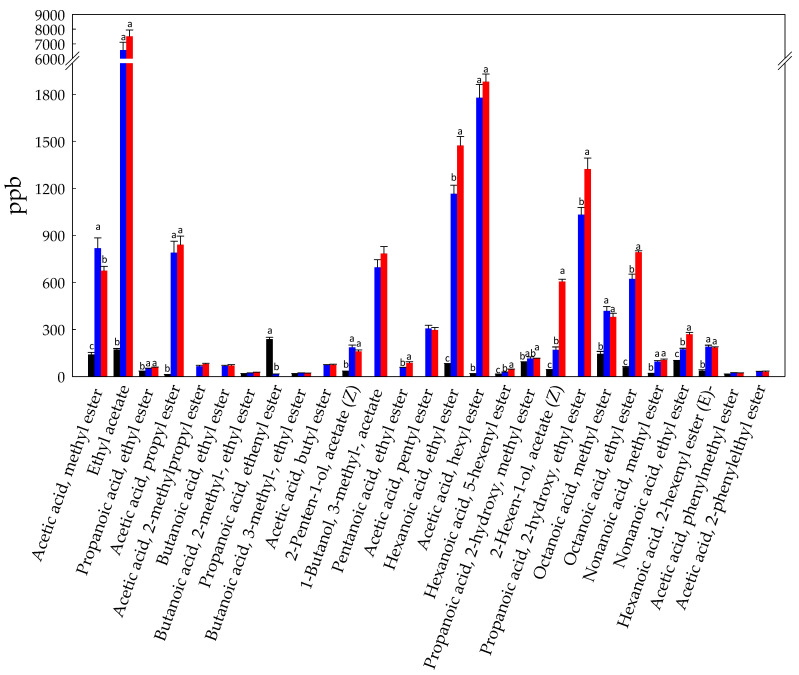
Concentration (ppb) of esters identified in Raw- (black bars), Unstarted- (blue bars), and Started-BCP (red bars). Data are the means (± SD) of three independent experiments analyzed in triplicate. Data were subjected to one-way ANOVA; pair-comparisons of treatment means was achieved by Tukey’s procedure at *p* < 0.05. For each compound, bars with different superscript letters differ significantly (*p* < 0.05).

Esters are flavor-active compounds that generally result in desirable fruity and floral notes in fermented foods [37]. In Raw-BCP, esters were mainly represented by propanoic acid ethenyl ester (237 ± 13 ppb), ethyl acetate (172 ± 7 ppb), octanoic acid methyl ester (144 ± 15 ppb), and acetic acid methyl ester (141 ± 13 ppb) (Figure 5). The noticeable increase in the level of esters found in fermented BCP (Figure 5), and especially in Started-BCP, may be associated with the higher availability of the alcohol precursors. In fact, the biosynthesis of esters in food systems proceeds through the reactions, catalyzed by microbial esterase or acyltransferase, between alcohols and carboxylic acids or between alcohols and acyl-CoA molecules [52]. Ethyl acetate was the main ester detected in Unstarted- and Started-BCP (6574 ± 535 and 7515 ± 426 ppb, respectively) (Figure 5), and usually represents a good marker of non-*Saccharomyces* species (e.g., *Hanseniaspora* spp.) during foods fermentations [53]. Hexanoic acid ethyl ester, acetic acid hexyl ester, and propanoic acid 2-hydroxy ethyl ester were also highly represented in fermented BCP samples (Figure 5). Esters play an important role in the flavor of fermented BCP, accounting for most of the VOC, with VFFA and alcohols.

**Figure 6:**
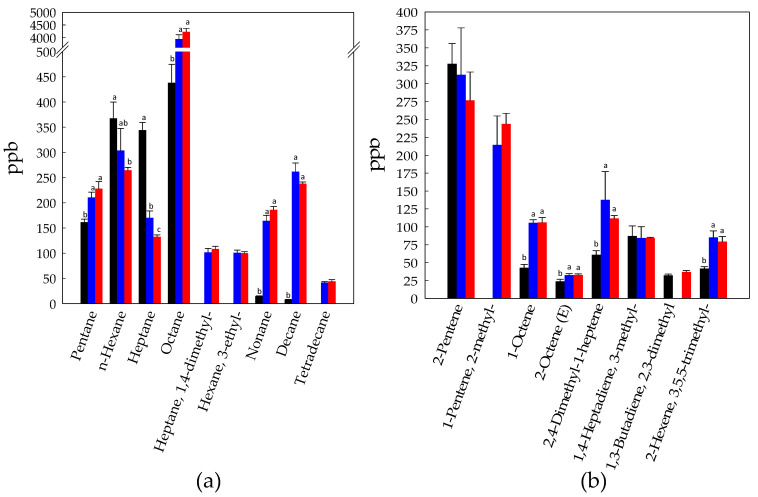
Concentration (ppb) of alkanes (**a**) and alkenes (**b**) identified in Raw- (black bars), Unstarted- (blue bars), and Started-BCP (red bars). Data are the means (± SD) of three independent experiments analyzed in triplicate. Data were subjected to one-way ANOVA; pair-comparisons of treatment means was achieved by Tukey’s procedure at *p* < 0.05. For each compound, bars with different superscript letters differ significantly (*p* < 0.05).

Hexane, heptane, octane and pentane were the main alkanes detected in Raw-BCP (Figure 6a). During fermentation, the levels of octane and, to a lesser extent of pentane, significantly increased (*p* < 0.05), whereas the concentrations of hexane and heptane decreased (*p* < 0.05). Nonane and decane, which were found in small amounts in Raw-BCP, increased during fermentation; 1,4-dimethyl-heptane, 3-ethyl-hexane, and tetradecane were detected only in fermented BCP samples (Figure 6a). Alkenes were also found at higher levels in Started and Unstarted-BCP with respect to Raw-BCP (Figure 6b).

Terpenes were poorly represented in Raw-BCP, with only 4 compounds detected at low concentrations: d-limonene (16 ± 1 ppb), cis-linalool oxide (34 ± 3 ppb), α-Linalool (15 ± 1), and cis-geraniol (80 ± 7 ppb). During fermentation, α-linalool significantly increased in Started-BCP (ca. 400%), and, to a lesser extent, in Unstarted-BCP (ca. 200%). The amount of d-limonene increased in all fermented samples (ca. 100%). The increase of free terpene levels was attributable to the release of glycosidically conjugated precursors, as a consequence of enzymatically (β-glucosidase) or acid hydrolyses, resulting in enhanced flavoring and functional properties [54]. Terpenes are usually correlated to pine, citrus and spicy notes [37]. Natural monoterpenes as well as their synthetic derivatives were reported to have various health-promoting features. Antioxidant properties were attributed to limonene and linalool, which act as termination enhancing antioxidants. Their activity it is not linearly related to their concentration, and their effectiveness depends on the rate of chain-termination of the oxidizable substrate [34]. Anti-inflammatory, vaso-relaxation and sedative effects were also reported for linalool [55,56,57]. Anti-inflammatory activity of linalool was associated to the downregulation of pro-inflammatory mediators and to the modulation of NF-kB and MAPKs signaling pathways. Sedative and anesthetics effects were linked to the interference with nicotinic acetylcholine and γ-aminobutyric acid type A receptors by linalool. Vaso-relaxation activity was associated to the activation of soluble guanylyl cyclase and K^+^ channels.

Several VOC, whose concentration increased during BCP fermentation, were previously shown to exert antimicrobial activities. For instance, limonene, phenyl ethyl alcohol, and 2-heptanone were shown to exert antibacterial or antifungal activities [32,33,58]. Focusing on antifungal activity, these compounds are able to damage the structure of fungal conidia, to prevent their germination, and to induce the down-regulation of gene expression involved in mycotoxins biosynthesis. Likely, such compounds contribute, together with organic acids, to the antimicrobial features recognized to lab scale-fermented BCP [13].

To our knowledge, few reports previously characterized the volatilome of bee bread or fermented BCP. Among the most complete studies, that of Kaškonienė et al. [59] identified dimethyl sulphide, acetic acid, furfural, nonane, 1-heptadecene, dodecane, 2,4-dimethylheptane, and hexanoic acid as preeminent compounds in multifloral bee bread. At a glance, only the high levels of some VFFA (acetic acid and hexanoic acid) and nonane were shared by both BCP fermented under the conditions of our study and beebread previously characterized. Our findings were partially discordant with that of Mayda et al. [60], who compared the fatty acid profiles of bee pollen and bee bread samples from the same bee hive. According to their report, the VFFA content in bee bread was not higher than found in fresh bee pollen, except for one sample showing higher level of octanoic acid. We have to point that a systematic and ultimate comparison between bee bread and lab-scale fermented BCP is difficult to achieve, because bee bread usually present traces of other interfering substances (e.g., honey). Furthermore, bee bread maturation is susceptible to instability and variations due to the diversity of the associated microbiota, the botanical origin of pollen, and the climatic conditions.

### 3.2. In Vitro Gastrointestinal Batch Digestion of BCP and Phenolics Bioaccessibility Assay

BCP represents a valuable source of phenolic compounds, with flavonoids as the most represented class, followed by phenolic acids [61]. A plethora of biological activities have been recognized to BCP phenolics [62], but their bioaccessibility is strongly limited by the pollen grain wall, that is not easily digestible by mono-gastric organisms, such as human [3,4,5]. Furthermore, bioaccessibility of phenolic compounds depends on their chemodiversity. Low bioaccessibility of phenolics may represent a critical point for using BCP as dietary supplement. Since fermentation was previously shown to improve the digestibility of BCP [13], we investigated how controlled or spontaneous fermentation may affect the profile of bioaccessible phenolic. In addition to the changes in the integrity of the pollen wall, fermentation may lead to structural and chemical changes of phenolics, which may result in modified bioaccessibility and bioactivity. Even though evidences resulting from metabolomic approaches are lacking, some authors previously reported the increase of total free phenolics level and antioxidant activity in bee pollen having undergone fermentation under controlled conditions [13,63,64]. Similarly, structural modification of the pollen grain with a consequent improved extractability of phenolics was also achieved after high pressure treatments [65]. The literature appears in agreement about the potential of lab-scale fermentation to promote the release of phenolic compounds from BCP [13,63,64]. On the other hand, the evaluations regarding the increase of free phenolics level during bee bread maturation are conflicting. According to Tomàs et al. [66], bee bread presents higher level of phenolics compared to raw BCP. On the contrary, Zuluaga-Dominguez & Quicazan [63] found comparable phenolic content in bee bread and raw BCP, whereas Mayda et al. [60] found lower levels of phenolics in bee bread compared to raw BCP. To our opinion, such discrepancies are due to the variability of bee bread maturation process.

**Figure 7:**
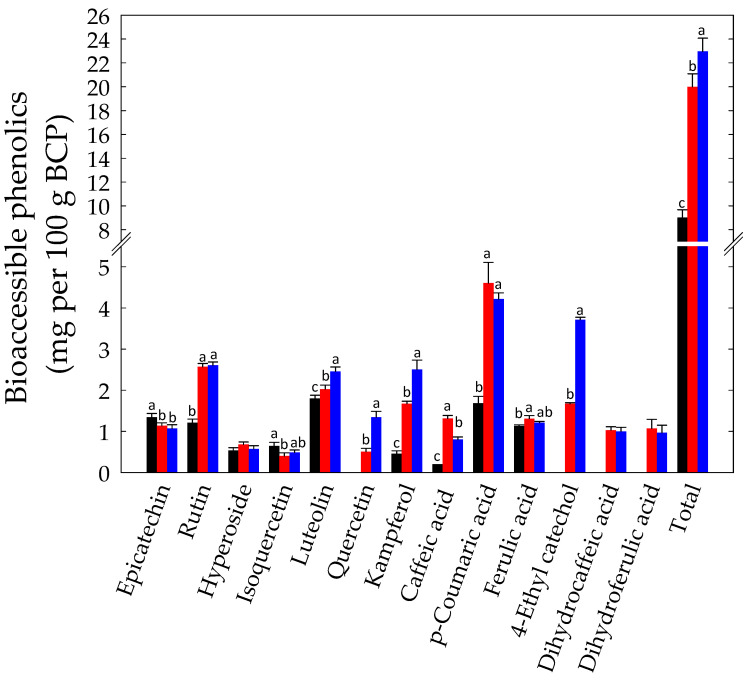
Bioaccessible phenolic compounds (mg per 100 g BCP) identified in Raw- (black bars), Unstarted- (blue bars), and Started-BCP (red bars). Total phenolics were calculated as the sum of individual compounds as detected by HPLC/UHPLC-ESI-MS/MS. Data are the means (± SD) of three independent experiments analyzed in triplicate. Data were subjected to one-way ANOVA; pair-comparison of treatment means was achieved by Tukey’s procedure at *p* < 0.05. For each compound, bars with different superscript letters differ significantly (*p* < 0.05).

Under the conditions of our study, nine compounds were identified into the bioaccessible fraction obtained from Raw-BCP, including phenolic acids and flavonoids that are known to be present in bee pollen (Figure 7) [67]. The bioaccessibility of many of these compounds increased following BCP fermentation. With respect to Raw-BCP, rutin and *p*-coumaric acid were found at higher level (*p* < 0.05) into the bioaccessible fraction of Unstarted- and Started-BCP (Figure 7). Luteolin and kampferol were also more bioaccesible (*p* < 0.05) after digestion of Started-BCP, and to a lesser extent, of Unstarted-BCP (Figure 7). An increase of caffeic acid bioaccessibility was found for Started-BCP, and especially, Unstarted-BCP. On the other side, quercetin, 4-ethyl catechol, dihydrocaffeic acid, and dihydroferulic acid were only found after digestion of fermented BCP samples, in particular of Started-BCP (Figure 7). Although the profile and the bioaccessibility of phenolics compounds is strongly dependent on the botanical origin of BCP, fermentation clearly enhanced the release of phenolics associated to carbohydrates, proteins, or to the pollen wall, and converted phenolics into different forms. Accordingly, the highest increase into the bioaccessible fraction was observed for *p*-coumaric acid, which is a structural component of sporopollenin, the principal matrices comprising the outer wall of pollen [68,69]. In particular, yeasts may have a prominent role in degradation of the plant cell wall and in release of non-extractable phenolics [13,26]. Yeast and/or lactic acid bacteria contributed also to increase bio-accessibility of non-extractable phenolics like proanthocyanidins, rutin, or luteolin by the degradation of associated proteins and carbohydrates [70,71]. Furthermore, during plant fermentations, phenolics are converted to biologically active metabolites through glycosyl hydrolase, phenolic acid decarboxylase and reductase, and esterase activities, which have been previously described also in lactic acid bacteria and yeasts [47,72,73,74]. The higher content of epicatechin after gastrointestinal digestion might be due to the partial hydrolysis of proanthocyanidins [75]. Quercetin may be released by glycosides like rutin, through microbial glycosyl hydrolases. Microbial esterases acids likely underwent the release of caffeic acid, through the hydrolyses of esters of phenolic acids like chlorogenic acid [19].

Other than the increased nutrient bioaccessibility, the beneficial effect of fermentation might also be related to the microbial catabolites resulting from phenolics metabolism [19]. Hydroxycinnamic acids (for example, caffeic and ferulic acids) may be decarboxylated to the corresponding vinyl derivatives (for example, dihydrocaffeic and dihydroferulic acids) by lactic acid bacteria. These latter also display phenolic acid reductase activities, able to hydrogenate the double bond of hydroxycinnamic acids and their vinyl derivatives [19,47]. Hydroxycinnamate decarboxylase and vinyl phenol reductase activities have been also described in some yeasts [73,74]. Certain of these metabolites have improved biological and antioxidant activities compared to their precursors [19]. Dihydrocaffeic acid, the reduced derivative of caffeic acid, has well-known and higher antioxidant activity than its precursor [76,77]. The reduction of vinylcatechol led to ethyl catechol, which is able to interfere with the induction of NF-kB and MAPKs signaling pathways in mammalian cells [19]. Although both spontaneous and controlled fermentation affected the profile of bioaccessible phenolic, the selected microbial starters led to more substantial results.

## 4. Conclusions

BCP fermentation may imply complex microbial dynamics and may occur through a plethora of metabolic pathways, which strongly affect sensory and nutritional features of BCP. Offering a complete metabolomic framework, we showed how spontaneous or controlled fermentation might strongly modify the inherent physical, chemical, and functional attributes of BCP. Lab-scale fermentation of BCP resulted in increased levels of VFFA, esters and alcohols, and reduced levels of aldehydes compared to raw-BCP. Although dedicated information on fermented BCP is lacking in literature, such metabolic footprint is usual in several fermented plant matrices [37]. Esters and alcohols are generally associated with desirable fruity and floral notes in fermented plant foods, thus fermentation may positively affect consumers’ acceptability of BCP. Fermentation also led to a moderate release of some terpenes (α-linalool, d-limonene) with functional features. Microbial metabolic activity dramatically impacted the composition of phenolics content and their bioaccessibility. Fermented BCP was enriched in bioaccessible phenolics with recognized bioactivity in humans. In particular, rutin, luteolin, kampferol, quercetin, *p*-coumaric acid, and caffeic acid resulted from the breakdown of complex structures or molecules, whereas 4-ethyl catechol, dihydrocaffeic acid, and dihydroferulic acid resulted from the microbial metabolism involving phenolic acids.

Furthermore, we highlighted how the effects of fermentation are highly dependent on the involved microorganisms and on their phenotypes. BCP fermentation by the selected consortium composed of *A. kunkeei* and *H. uvarum* strains was more effective than spontaneous fermentation in releasing bioactive compounds, thus representing a valuable tool for a standardized production of fermented BCP with highly accessible bioactive compounds. On the other hand, mixed fermentation by lactic acid bacteria and yeast allowed overcoming the limits of single strain fermentation during BCP processing [13,63].

In light of the prominent role that selected microbial starters have during BCP fermentation, our study may be used as a starting point to select ad-hoc microbial starters for improved flavors and human health-related features of BCP. For instance, our detailed insight into microorganisms–substrates interactions may be used to identify target phenotypes for microbial strains selection. In any case, further investigations based on integrated metabolomic and metagenomic approaches will be useful to provide more in depth understanding of microbial dynamics and to fully exploit the wide enzymatic reservoir of lactic acid bacteria and yeasts for BCP processing.

## Data Availability

Not applicable.

## Supplementary Materials

The following are available online at https://www.mdpi.com/2304-8158/10/2/286/s1, Table S1: Mass transitions (MRM) and instrumental parameters optimized for each phenolic compound for the analysis by LC-ESI-MS/MS, Table S2: Intra-day and inter-day (CV%), recovery (%), accuracy (%RE) and precision (%RSD), Table S3: Volatile compounds (ppb) identified in Raw-, Unstarted-, and Started-BCP. Data are the means (± SD) of three independent experiments analyzed in triplicate.
