# Supplementary material for: Volatilome and Bioaccessible Phenolics Profiles in Lab-Scale Fermented Bee Pollen
Source: Foods. 2021 Jan 31;10(2):286. doi: 10.3390/foods10020286 (PMC7911640; doi:10.3390/foods10020286)
Supplement: Supplementary file 1 [file foods-10-00286-s001.zip › foods-1056735 - Supplementary files/Table S1.docx]

**Table S1.** Mass transitions (MRM) and instrumental parameters optimized for each phenolic compound for the analysis by LC-ESI-MS/MS.

| **Compound** | **Ionization Mode** | **Precursor ion** | **Q1** | |  | **Q2** | |  | **Q2** | | **RT (min)** |
| --- | --- | --- | --- | --- | --- | --- | --- | --- | --- | --- | --- |
|  |  |  | **Product ion Q1** | **Collision energy (V)** |  | **Product ion Q2** | **Collision energy (V)** |  | **Product ion Q2** | **Collision energy (V)** |  |
| Caffeic acid | - | 179.1 | 135.1 | 17 |  | 134.1 | 28 |  | 89.2 | 44 | 5.54 |
| Epicatechin | - | 289.2 | 245 | 17 |  | 203.1 | 21 |  | 123.1 | 29 | 6.38 |
| *p*-Coumaric acid | + | 165.1 | 119.2 | 16 |  | 91.2 | 25 |  | 65.3 | 33 | 6.31 |
| Rutin | - | 609.3 | 343 | 31 |  | 299 | 39 |  |  |  | 6.67 |
| Hyperoside | - | 463.2 | 300.1 | 26 |  | 271.1 | 21 |  |  |  | 6.87 |
| Ferulic acid | + | 195 | 145 | 50 |  | 117 | 24 |  |  |  | 6.93 |
| Isoquercetin | - | 463.1 | 300 | 42 |  | 270.9 | 26 |  |  |  | 6.97 |
| Luteolin | + | 287 | 153 | 32 |  | 135 | 30 |  |  |  | 9.31 |
| Quercetin | + | 303 | 229.1 | 27 |  | 153.1 | 32 |  |  |  | 9.37 |
| Kampferol | + | 287 | 165 | 26 |  | 153 | 31 |  |  |  | 10.31 |
|  |  |  |  |  |  |  |  |  |  |  |  |
