# Supplementary material for: Volatilome and Bioaccessible Phenolics Profiles in Lab-Scale Fermented Bee Pollen
Source: Foods. 2021 Jan 31;10(2):286. doi: 10.3390/foods10020286 (PMC7911640; doi:10.3390/foods10020286)
Supplement: Supplementary file 1 [file foods-10-00286-s001.zip › foods-1056735 - Supplementary files/Table S2.docx]

**Table S2.** Intra-day and inter-day (CV%), recovery (%), accuracy (%RE) and precision (%RSD)*.

| **Compound** | **Intra-day CV (%)** | **Inter-day CV (%)** | **Recovery** | **Accuracy** | **Precision** |
| --- | --- | --- | --- | --- | --- |
| Caffeic acid | 6.30 | 14.49 | 100 | 38 | 5 |
| Epicatechin | 3.71 | 14.81 | 79 | -35 | 3 |
| *p*-Coumaric acid | 6.21 | 18.38 | 111 | 25 | 3 |
| Rutin | 3.30 | 18.40 | 102 | 3 | 1 |
| Hyperoside | 10.36 | 9.03 | 93 | 28 | 6 |
| Ferulic acid | 4.46 | 18.36 | 106 | 11 | 5 |
| Isoquercetin | 3.30 | 5.51 | 73 | 30 | 6 |
| Luteolin | 4.37 | 15.37 | 75 | -4 | 3 |
| Quercetin | 6.19 | 18.66 | 92 | -2 | 3 |
| Kampferol | 5.20 | 9.49 | 95 | -9 | 3 |

*Intra-day and inter-day parameters underwent calculation by injecting 10 times a QC sample at middle concentration level on the same day and re-injecting for 6 consecutive days. Intra- and inter-day variability were evaluated by coefficients of variation (CV%). The recovery test was carried out to assess the applicability of the LC-ESI-MS/MS technique, and it was determined as the average of the "measured value" / "the expected value" ratio (%). Precision values were calculated as Relative Standard Deviation (%RSD), among the measures replicated in the QC sample, which were obtained by analyzing ten times the same sample. Since the precision may vary with the concentration, it was appropriate to analyze at least three samples at different concentrations (low, medium and high) compared to the calibration range for each analyte. The accuracy was calculated as the difference between the calculated value and the theoretical value divided by the theoretical value, re-expressed as the Relative Error percentage (%RE).
