# Supplementary material for: Volatilome and Bioaccessible Phenolics Profiles in Lab-Scale Fermented Bee Pollen
Source: Foods. 2021 Jan 31;10(2):286. doi: 10.3390/foods10020286 (PMC7911640; doi:10.3390/foods10020286)
Supplement: Supplementary file 1 [file foods-10-00286-s001.zip › foods-1056735 - Supplementary files/Table S3.docx]

**Table S3.** Volatile compounds (ppb) identified in Raw-, Unstarted-, and Started-BCP. Data are the means (± SD) of three independent experiments analyzed in triplicate.

|  | **ppb** | | |
| --- | --- | --- | --- |
|  | **Raw-BCP** | **Unstarted-BCP** | **Started-BCP** |
| **Volatile free fatty acids** |  |  |  |
| Decanoic acid, 2-methyl- | 173 ± 11 | 324 ± 22 | 310 ± 16 |
| Acetic acid | 1,246 ± 122 | 53,475 ± 2391 | 47,990 ± 1399 |
| Propionic acid | 150 ± 18 | 148 ± 9 | 132 ± 8 |
| Butanoic acid | 52 ± 6 | 105 ± 5 | 104 ± 4 |
| Butanoic acid, 4-hydroxy- | 102 ± 8 | 92 ± 7 | 87 ± 6 |
| Butanoic acid, 3-methyl- | 57 ± 4 | 228 ± 14 | 242 ± 17 |
| Pentanoic acid | 15 ± 1 | 42 ± 1 | 45 ± 5 |
| Hexanoic acid | 204 ± 13 | 496 ± 47 | 497 ± 59 |
| Heptanoic acid | 6 ± 0 | 26 ± 6 | 31 ± 5 |
| Octanoic acid | 67 ± 3 | 209 ± 37 | 182 ± 2 |
| Nonanoic acid | 32 ± 3 | 85 ± 7 | 85 ± 4 |
| **Alkanes** |  |  |  |
| Pentane | 162 ± 6 | 210 ± 11 | 228 ± 14 |
| n-Hexane | 368 ± 32 | 304 ± 44 | 264 ± 6 |
| Heptane | 344 ± 15 | 170 ± 14 | 133 ± 4 |
| Octane | 438 ± 36 | 3,943 ± 168 | 4,228 ± 130 |
| Heptane, 1,4-dimethyl- | n.d. | 102 ± 8 | 108 ± 5 |
| Hexane, 3-ethyl- | n.d. | 101 ± 5 | 100 ± 3 |
| Nonane | 15 ± 1 | 164 ± 11 | 186 ± 7 |
| Decane | 8 ± 1 | 262 ± 18 | 238 ± 4 |
| Tetradecane | n.d. | 42 ± 2 | 44 ± 4 |
| **Alkenes** |  |  |  |
| 2-Pentene | 328 ± 28 | 312 ± 66 | 277 ± 40 |
| 1-Pentene, 2-methyl- | n.d. | 214 ± 40 | 244 ± 15 |
| 1-Octene | 43 ± 5 | 106 ± 4 | 106 ± 7 |
| 2-Octene (E) | 24 ± 3 | 32 ± 2 | 33 ± 1 |
| 2,4-Dimethyl-1-heptene | 61 ± 6 | 138 ± 40 | 112 ± 4 |
| 1,4-Heptadiene, 3-methyl- | 87 ± 14 | 84 ± 16 | 84 ± 1 |
| 1,3-Butadiene, 2,3-dimethyl | 32 ± 2 | n.d. | 37 ± 2 |
| 2-Hexene, 3,5,5-trimethyl- | 42 ± 3 | 85 ± 9 | 79 ± 7 |
| **Alcohols** |  |  |  |
| Methanol | 57 ± 1 | 184 ± 12 | 194 ± 6 |
| 2-Propanol | 70 ± 6 | 40 ± 4 | 41 ± 4 |
| Ethanol | 576 ± 53 | 3,212 ± 181 | 4,120 ± 90 |
| 1-Propanol | 147 ± 12 | 251 ± 10 | 329 ± 29 |
| 3-Pentanol | 48 ± 13 | 72 ± 4 | 108 ± 47 |
| 1-Pentyn-3-ol | 102 ± 4 | n.d. | n.d. |
| 1-Penten-3-ol | 1,101 ± 74 | 581 ± 23 | 556 ± 19 |

**Table S3.** Continued.

|  | **ppb** | | |
| --- | --- | --- | --- |
|  | **Raw-BCP** | **Unstarted-BCP** | **Started-BCP** |
| **Alcohols** |  |  |  |
| 1-Butanol, 3-methyl- | 163 ± 10 | 316 ± 15 | 343 ± 14 |
| 1-Pentanol | 96 ± 8 | 289 ± 13 | 265 ± 11 |
| Undecanal | 133 ± 13 | 79 ± 7 | 77 ± 5 |
| 2-Penten-1-ol (Z) | 20 ± 2 | 65 ± 5 | 46 ± 2 |
| 1-Hexanol | 813 ± 42 | 971 ± 41 | 1,101 ± 50 |
| 2-Hexen-1-ol, (E) | 23 ± 0 | 79 ± 10 | 77 ± 6 |
| 4-Penten-2-ol | n.d. | 79 ± 7 | 92 ± 8 |
| 1-Octen-3-ol | 168 ± 14 | 207 ± 3 | 214 ± 5 |
| 1-Heptanol | 35 ± 2 | 144 ± 16 | 170 ± 13 |
| n-Tridecan-1-ol | 31 ± 4 | 150 ± 9 | 208 ± 15 |
| 1-Octanol | 38 ± 4 | 35 ± 2 | 56 ± 6 |
| 1,5,7-Octatrien-3-ol, 3,7-dimethyl- | n.d. | 71 ± 3 | 67 ± 1 |
| 6-Hepten-1-ol, 2-methyl- | 31 ± 2 | 84 ± 5 | 68 ± 2 |
| 1-Nonanol | 28 ± 1 | 785 ± 55 | 914 ± 17 |
| Benzyl alcohol | 32 ± 2 | 69 ± 6 | 84 ± 9 |
| Phenylethyl alcohol | 64 ± 5 | 143 ± 13 | 132 ± 7 |
| **Aldehydes** |  |  |  |
| Propanal | 230 ± 11 | n.d. | n.d. |
| Butanal, 2-methyl- | 25 ± 2 | 18 ± 4 | 21 ± 2 |
| Hexanal | 3650 ± 91 | 3 ± 0 | 97 ± 3 |
| Nonanal | 633 ± 37 | 301 ± 25 | 325 ± 31 |
| 3-Furaldehyde | n.d. | 67 ± 5 | 250 ± 19 |
| 2,4-Heptadienal (E,E) | 813 ± 31 | 50 ± 8 | 59 ± 2 |
| **Ketons** |  |  |  |
| Acetone | 65 ± 7 | 92 ± 4 | 82 ± 2 |
| 2-Butanone | 21 ± 2 | 64 ± 4 | 61 ± 5 |
| 3-Pentanone | 82 ± 7 | 321 ± 27 | 322 ± 25 |
| 2-Heptanone | 62 ± 2 | 272 ± 17 | 224 ± 11 |
| Cyclopentanone, 2 methyl | 397 ± 10 | 47 ± 3 | 25 ± 4 |
| 2-Heptanone, 6-methyl- | 28 ± 2 | 103 ± 4 | 85 ± 4 |
| 3-Octanone | 56 ± 2 | 188 ± 8 | 198 ± 5 |
| 6-Octen-2-one (Z) | 56 ± 3 | 52 ± 4 | 91 ± 3 |
| 5-Hepten-2-one, 6-methyl- | 1,244 ± 121 | 2,158 ± 92 | 1,735 ± 57 |
| 3,5-Octadien-2-one | 719 ± 25 | 99 ± 2 | 83 ± 6 |
| 3,5-Heptadien-2one, 6-methyl-(E) | 7 ± 1 | 77 ± 3 | 55 ± 1 |
| 2-Cyclohexen-1-one, 3,5,5-trimethyl- | 59 ± 4 | 153 ± 3 | 112 ± 2 |
| **Esters** |  |  |  |
| Acetic acid, methyl ester | 141 ± 13 | 819 ± 66 | 675 ± 28 |
| Ethyl acetate | 172 ± 7 | 6,574 ± 535 | 7,515 ± 426 |
| Propanoic acid, ethyl ester | 33 ± 1 | 49 ± 5 | 57 ± 3 |

**Table S3.** Continued.

|  | **ppb** | | |
| --- | --- | --- | --- |
|  | **Raw-BCP** | **Unstarted-BCP** | **Started-BCP** |
| **Esters** |  |  |  |
| Acetic acid, propyl ester | 12 ± 1 | 790 ± 73 | 840 ± 55 |
| Acetic acid, 2-methylpropyl ester | n.d. | 66 ± 7 | 79 ± 7 |
| Butanoic acid, ethyl ester | n.d. | 66 ± 6 | 69 ± 7 |
| Butanoic acid, 2-methyl-, ethyl ester | 19 ± 0 | 22 ± 1 | 26 ± 2 |
| Propanoic acid, ethenyl ester | 237 ± 13 | 15 ± 1 | ± |
| Butanoic acid, 3-methyl-, ethyl ester | 18 ± 2 | 22 ± 2 | 21 ± 1 |
| Acetic acid, butyl ester | n.d. | 72 ± 5 | 73 ± 5 |
| 2-Penten-1-ol, acetate (Z) | 31 ± 3 | 186 ± 14 | 160 ± 12 |
| 1-Butanol, 3-methyl-, acetate | n.d. | 697 ± 49 | 784 ± 45 |
| Pentanoic acid, ethyl ester | n.d. | 57 ± 2 | 87 ± 7 |
| Acetic acid, pentyl ester | n.d. | 306 ± 20 | 296 ± 16 |
| Hexanoic acid, ethyl ester | 80 ± 3 | 1,165 ± 55 | 1,473 ± 58 |
| Acetic acid, hexyl ester | 18 ± 1 | 1,778 ± 85 | 1,882 ± 49 |
| Hexanoic acid, 5-hexenyl ester | 14 ± 1 | 27 ± 2 | 45 ± 3 |
| Propanoic acid, 2-hydroxy, methyl ester | 92 ± 10 | 114 ± 12 | 114 ± 3 |
| 2-Hexen-1-ol, acetate (Z) | 42 ± 2 | 170 ± 18 | 606 ± 15 |
| Propanoic acid, 2-hydroxy, ethyl ester | n.d. | 1,032 ± 47 | 1,323 ± 71 |
| Octanoic acid, methyl ester | 144 ± 15 | 417 ± 29 | 380 ± 22 |
| Octanoic acid, ethyl ester | 59 ± 6 | 623 ± 30 | 792 ± 9 |
| Nonanoic acid, methyl ester | 20 ± 1 | 95 ± 6 | 105 ± 6 |
| Nonanoic acid, ethyl ester | 101 ± 2 | 173 ± 6 | 269 ± 11 |
| Hexanoic acid. 2-hexenyl ester (E)- | 36 ± 6 | 190 ± 10 | 183 ± 6 |
| Acetic acid, phenylmethyl ester | 15 ± 5 | 22 ± 2 | 22 ± 2 |
| Acetic acid, 2-phenylelthyl ester | n.d. | 32 ± 2 | 30 ± 4 |
| **Furans** |  |  |  |
| Furan, 2-ethyl- | 133 ± 12 | 194 ± 9 | 186 ± 8 |
| Trans-2-(2-pentenyl)furan | 38 ± 11 | 203 ± 11 | 189 ± 5 |
| 3-Furanmethanol | 9 ± 1 | 78 ± 2 | 58 ± 3 |
| **Lacton** |  |  |  |
| 2(3H)-Furanone, dihydro-5-methyl- | 48 ± 3 | 50 ± 3 | 47 ± 1 |
| **Terpenes** |  |  |  |
| d-Limonene | 16 ± 1 | 37 ± 3 | 36 ± 1 |
| cis-Linalool oxide | 34 ± 3 | 37 ± 2 | 38 ± 6 |
| α-Linalool | 15 ± 1 | 44 ± 3 | 74 ± 7 |
| cis-Geraniol | 80 ± 7 | 67 ± 6 | 68 ± 6 |
| **Sulphur compound** |  |  |  |
| Dimethyl sulfide | 117 ± 4 | 253 ± 50 | 208 ± 4 |

n.d., not detected.
